# Extensively Drug-Resistant *Mycobacterium tuberculosis*, India

**DOI:** 10.3201/eid1309.070443

**Published:** 2007-09

**Authors:** Rajesh Mondal, Amita Jain

**Affiliations:** * King George’s Medical University, Lucknow, India

**Keywords:** XDR TB, Mycobacterium tuberculosis, India, letter

**To the Editor:** India is contributing nearly one third of the world’s tuberculosis (TB) cases and has the highest rate of new TB cases (*1*). Prevalence of multidrug-resistant TB (MDR TB) cases is on the rise in India, and proportions of new cases of MDR TB have been observed to vary from 1.1% to 5.3% in most of the reported studies. The proportion of previously treated patients with MDR TB varied from 8% to 67% (*2*). Although these studies have been conducted in different parts of India, they indicate an increasing trend of MDR TB cases.

MDR TB cases threaten the effectiveness of chemotherapy for both treatment and control of TB and require the use of second-line drugs that are more expensive, toxic, and less effective than first-line anti-TB drugs (*3*). The Green Light Committee established by the Stop TB partners (*4*), which ensures the proper use of second-line drugs to prevent increasing drug resistance in MDR TB cases in resource-limited countries, encountered resistance to these drugs. This led to the emergence of new terminology in relation to drug-resistant TB, i.e., extensively drug-resistant TB (XDR TB). XDR TB is defined as TB caused by a *Mycobacterium tuberculosis* strain that is resistant to at least rifampin and isoniazid among the first-line anti-TB drugs (MDR TB) in addition to resistance to any fluoroquinolones and at least 1 of 3 injectable second-line drugs (*5*). A recent report describes the current prevalence of XDR TB worldwide (*6*). Although India has high annual risk for TB cases and increasing prevalence of MDR TB cases, XDR TB has not yet been described in India.

From December 2000 through December 2002, 68 MDR TB isolates were obtained from sputum samples from pulmonary TB patients, referred to Department of Microbiology, King George’s Medical University, Lucknow Uttar Pradesh, India, for culture and sensitivity testing. Drug susceptibility testing for first-line drugs was performed by 1% proportion method against streptomycin (4 μg/mL), isoniazid (0.2 μg/mL), rifampin (40 μg/mL), and ethambutol (2 μg/mL) (*7*). The susceptibility of MDR TB isolates against second-line drugs was done by the absolute concentration method (MIC) for ofloxacin (0.5–16 μg/mL) and kanamycin (2–64 μg/mL) and by 1% proportional sensitivity method for ethionamide (40 μg/mL), p-amino salicylic acid (0.5 μg/ml), clarithromycin (2 μg/mL), and capreomycin (40 μg/mL). Resistance to ofloxacin, kanamycin, and ethionamide was determined by a cut-off of MIC >8 μg/mL, >64 μg/mL, and >128 μg/mL, respectively (*8*). All drugs were procured from Sigma (St. Louis, MO, USA), and quality control for drug susceptibility test was provided by the Tuberculosis Research Center, Chennai, India.

Among 68 MDR strains, 21 were from patients who had never been previously treated, and 47 were from patients whose medical history was positive for anti-tubercular treatment in the past, for at least 4 weeks. All MDR TB isolates were tested for susceptibility to second-line drugs, and high resistance to these drugs was found. MDR strains, which were further resistant to ofloxacin, and to at least 1 of 2 injectable second-line drugs tested (i.e., kanamycin or capreomycin), were classified as XDR TB. A total of 5 (7.4%) of 68 MDR TB strains met criteria for XDR TB. XDR TB isolates were usually resistant to almost all 4 first-line and second-line anti-TB drugs tested (Table). Global data on XDR TB are limited; however, a recent article reported that the problem of XDR TB is worldwide and includes a prevalence of 6.6% XDR TB cases in the studied countries (*6*). The Republic of Korea reports the maximum numbers of such cases, with 200 (15.4%) of 1,298 XDR TB strains tested from MDR TB patients included in the study. On December 1, 2006, World AIDS Day, South Africa reported more than 300 cases of XDR TB (*9*).

Here, we report, to our knowledge, the first XDR TB cases in India and the emergence of XDR TB in settings like India, where adequate monitoring of treatment regimens for MDR TB in TB control programs is difficult to implement due to a huge population and the high annual risk of acquiring TB is of great concern. A limitation to accurate detection of XDR TB is those existing tests for resistance to second-line drugs is not yet standardized and is less reproducible than results for first-line drugs (*10*). Access to management and treatment of MDR TB cases with second-line drugs, standardized methods, improved diagnostics, and quality assurance for susceptibility testing are needed to ensure reliable testing and the design of appropriate drug regimens.

Our study has some limitations, however. The data are not representative of the whole community and are limited to 1 hospital. Limited numbers of drugs were used in drug susceptibility testing, and the sample size is also not statistically adequate. A community-based, multicenter study, which includes all parts of the country and uses the full spectrum of drugs, is needed to describe the true prevalence of XDR TB in India.

**Table:** Resistance pattern of XDR TB isolates*

Strain no.	Resistant to first-line drugs	Resistant to second-line drugs
RM 55	S, H, R, E	K, O, CAP, CLA, PAS, ETH
RM 490	S, H, R, E	K, O, CAP, CLA, PAS, ETH
RM 552	S, H, R, E	K, O, CAP, CLA, PAS, ETH
RM 585†	S, H, R, E	K, O, CLA, PAS, ETH
RM 789	S, H, R, E	K, O, CAP, CLA, PAS, ETH

*n = 5; XDR TB, extensively drug-resistant tuberculosis; S, streptomycin; H, isoniazid; R, rifampin; E, ethambutol; K, kanamycin; O, ofloxacin; CAP, capreomycin; CLA, clarithromycin; PAS, p– amino salicylic acid; ETH, ethionamide. †Sensitive to capreomycin.
